# Social Determinants of Health Among Chinese Americans at Risk for Diabetes in a Mobile Diabetes Prevention Trial: Cross-Sectional Baseline Analysis

**DOI:** 10.2196/95295

**Published:** 2026-06-03

**Authors:** Nan Jiang, Jing Liu, Haili Song, Yanan Zhao, Huilin Li, Stella S Yi, Jeannette M Beasley, Lu Hu

**Affiliations:** 1 Department of Population Health, NYU Grossman School of Medicine, 180 Madison Avenue, New York, NY, 10016, United States, +1 (646) 501-3438; 2 Department of Medicine, NYU Grossman School of Medicine, New York, NY, United States; 3 Department of Nutrition and Food Studies, Steinhardt School of Culture, Education, and Human Development, New York University, New York, NY, United States

**Keywords:** prediabetes, diabetes, social determinants of health, Chinese American, mobile health

## Abstract

**Background:**

Prediabetes is common in the United States, and adverse social determinants of health (SDOH) are known to undermine diabetes prevention efforts. Chinese Americans experience a disproportionately high prevalence of prediabetes, yet the SDOH profiles of this population remain understudied.

**Objective:**

This study assessed SDOH among Chinese Americans at risk for diabetes and examined the associations between sociodemographic characteristics and SDOH barriers.

**Methods:**

We conducted a cross-sectional analysis of baseline survey data from the Integrating Cultural Aspects into Diabetes Education (INCLUDE) study, a randomized controlled trial of a culturally and linguistically tailored mobile diabetes prevention intervention for Chinese Americans. Participants at risk for diabetes were enrolled between April 2023 and June 2024 in New York City (N=150). Measures included in the analyses were a 14-item SDOH scale (range 0-14, with higher scores indicating more barriers) and sociodemographic characteristics. Due to the small frequencies of high SDOH scores, we collapsed the outcome into 5 categories (0, 1, 2, 3, and 4-14) to improve model stability. We first used univariable logistic regression models to examine associations between each sociodemographic factor (age, sex, years of US residence, English proficiency, education, marital status, employment status, and annual household income) and the collapsed SDOH category, followed by a multivariable ordinal regression model including all sociodemographic variables.

**Results:**

A total of 150 participants had a mean age of 49.9 (SD 12.6) years. Most were female (n=124, 82.7%), born outside the United States (n=149, 99.3%), and reported speaking English less than very well (n=132, 88.0%). Among respondents to the SDOH items (n=149), the mean SDOH score was 2.4 (SD 2.3), and 81.9% (n=122) reported at least 1 SDOH barrier. The three most frequently reported barriers were (1) the need to improve English proficiency, reading skills, or educational attainment (n=77, 51.7%); (2) experiences of racial discrimination (n=49, 32.9%); and (3) adverse housing conditions (n=38, 25.5%). After collapsing the original SDOH score, 27 (18.2%) participants had a score of 0, 39 (26.2%) had a score of 1, 23 (15.4%) had a score of 2, 27 (18.2%) had a score of 3, and 33 (22.1%) had scores of 4 to 14. In the multivariable analysis, female sex (vs male) was associated with higher SDOH score categories (odds ratio 3.83, 95% CI 1.65-9.16; *P*=.002).

**Conclusions:**

SDOH-related barriers were prevalent among Chinese Americans at risk for diabetes. Diabetes prevention efforts should incorporate routine SDOH screening and structured resource navigation or referral pathways, with particular attention to subgroups at higher risk, such as female individuals.

## Introduction

Prediabetes imposes a substantial and growing health and economic burden in the United States [1]. National medical costs attributable to prediabetes were estimated at US $25 billion in 2007 and US $44 billion in 2012, representing a 74% increase over 5 years [2,3]. Prediabetes is a state of impaired glucose regulation, with blood glucose levels above normal but below diagnostic thresholds for diabetes [4]. In 2023, an estimated 115.2 million US adults aged ≥18 years (43.5% of the adult population) had prediabetes [5], and approximately 5% to 10% of individuals with prediabetes progress to type 2 diabetes annually [4,6]. Therefore, preventive strategies targeting individuals with prediabetes are essential to reduce progression to diabetes.

Social determinants of health (SDOH) refer to the conditions in which people are born, grow, live, work, and age [7]. Healthy People 2030 organizes SDOH into five domains: (1) economic stability, (2) education access and quality, (3) health care access and quality, (4) neighborhood and built environment, and (5) social and community context [8]. Adverse SDOH (eg, housing instability, food insecurity, limited insurance coverage, and restricted access to health care) affect diabetes outcomes by posing barriers to effective self-management, including healthy diet, physical activity, medication adherence, and glucose monitoring [9-12]. Evidence shows that adverse SDOH are associated with an increased risk of diabetes [13-15].

Chinese Americans represent the largest Asian American ethnic group in the United States, with approximately 5.5 million individuals, including 2.9 million who were born outside the United States [16,17]. Chinese Americans also experience a disproportionately high prevalence of prediabetes. The burden of diabetes and prediabetes is substantial in this population. Among 373,098 adults aged 45 to 64 years in the Kaiser Permanente Northern California health plan, the prevalence of prediabetes was significantly higher among Chinese Americans than among White individuals (37.8% vs 24.5% among men; 30.8% vs 18.0% among women) [18]. Chinese Americans, particularly immigrants, often face SDOH barriers, including lower education and income, limited English proficiency, and a lack of health insurance [19-21]. These barriers may impede access to diabetes prevention programs and increase the risk of progression from prediabetes to diabetes.

Despite growing attention to SDOH in diabetes prevention and care, little is known about SDOH profiles among Chinese Americans at risk for diabetes. To address this gap, we conducted an exploratory cross-sectional analysis of baseline data from a randomized controlled trial of a culturally and linguistically tailored, digital diabetes prevention intervention for Chinese Americans at risk for diabetes. The objectives of this study were to describe SDOH status and assess associations between sociodemographic characteristics and SDOH barriers within this sample. The findings will provide insights into SDOH patterns in an understudied population and may inform future diabetes prevention efforts among Chinese Americans.

## Methods

### Study Design

We analyzed baseline survey data from the Integrating Cultural Aspects into Diabetes Education (INCLUDE) trial, a randomized controlled trial evaluating the efficacy of a culturally and linguistically tailored mobile diabetes prevention program for Chinese Americans at risk of diabetes, with weight loss as the primary outcome. Details about the INCLUDE trial have been reported elsewhere [22].

### Ethical Considerations

The study protocol was approved by the New York University Grossman School of Medicine Institutional Review Board (s22-00783) and was registered at ClinicalTrials.gov (NCT05492916). All participants were fully informed, both verbally and in writing, of the study purpose, procedures, potential risks and benefits, the voluntary nature of participation, and their right to withdraw. Participant privacy and confidentiality were strictly maintained throughout the study. Written informed consent was obtained from all participants prior to the baseline survey. Participants received a US $25 gift card as compensation for completing the baseline survey.

### Participants and Recruitment

A total of 150 participants residing in New York City (NYC) were enrolled in the INCLUDE trial between April 2023 and June 2024. Eligibility criteria were as follows: (1) self-identification as being of Chinese ethnicity; (2) aged 18 to 70 years; (3) diagnosis of prediabetes or a score of ≥5 on a validated prediabetes risk test [23]; (4) BMI ≥23 kg/m², using the Asian-specific overweight threshold [24]; (5) willingness to receive program videos via SMS text messaging or WeChat (Tencent Holdings Ltd); and (6) smartphone ownership or willingness to use a study-provided smartphone. Exclusion criteria were as follows: (1) inability or unwillingness to provide informed consent, (2) hearing or vision impairment that precluded participation, (3) refusal of randomization, and (4) current pregnancy or breastfeeding or plans to become pregnant within the next 6 months.

Participants were recruited using five primary strategies: (1) partnerships with community-based organizations and health care facilities serving Chinese American communities in NYC to disseminate study flyers (eg, posting flyers in offices, on organizational websites, and through WeChat Moments); (2) advertisements in local newspapers and other media outlets; (3) community outreach events (eg, food pantries, health fairs, and health workshops); (4) proactive outreach using the New York University Langone Health Epic electronic health record across Manhattan, Queens, Brooklyn, and Long Island (potentially eligible individuals were identified, mailed an invitation letter describing the study, and then contacted by phone); and (5) peer referrals, in which individuals voluntarily shared study flyers within their social networks without financial incentives.

### Screening, Enrollment, and Baseline Survey

Interested individuals contacted bilingual research assistants (RAs) by calling the study phone number or approaching RAs at in-person recruitment activities (eg, community outreach events). Trained RAs conducted eligibility screening by phone or in person. For eligible participants, RAs obtained informed consent (verbal consent for phone-based sessions and written consent for in-person sessions) and administered the baseline survey by phone or in person. Participants could complete the consent process and the survey in Chinese or English. Participants received the compensation after completing the baseline survey.

### Measures

The analysis included a 14-item SDOH measure and sociodemographic characteristics. The 14-item SDOH instrument was adapted from validated tools, including the brief food insecurity screener [25] and domains from the Accountable Health Communities Health-Related Social Needs Screening Tool [26]. This instrument underwent a standard translation and back-translation process to ensure linguistic accuracy and cultural appropriateness for this population. It assessed core health-related barriers and social needs across 5 domains, including economic stability (eg, food access and housing stability), education access and quality (eg, need to improve English proficiency, reading skills, or educational attainment), health care access and quality (eg, insurance coverage), neighborhood and built environment (eg, housing conditions), and social and community context (eg, racial discrimination; need for childcare; and need for help addressing tobacco, alcohol, or drug use). Items had dichotomous response options (1=“Yes” and 0=“No”). An overall SDOH score was calculated by summing the responses across the 14 items (range 0-14), with higher scores indicating more SDOH barriers. Given the relatively small sample size and the exploratory nature of the analysis, we used a composite score to capture the overall burden of SDOH barriers rather than performing domain-specific analyses to maintain statistical power.

Sociodemographic characteristics included age, sex (female or male), country of birth (born outside the United States vs born in the United States), years of residence in the United States, self-reported English proficiency (“very well,” “well,” “not well,” and “not at all”), education (less than high school, high school graduate, and more than high school), marital status (married or cohabitating vs other), employment status (employed, unemployed, and retired), annual household income (US <$25,000, US $25,000-$55,000, US >$55,000, and “unreported/don’t know”), and health insurance type (Medicaid, Medicare, private insurance, other public or government insurance, employer-sponsored insurance, and uninsured). For health insurance type, participants could select all applicable responses.

### Statistical Analysis

Descriptive statistics summarized sociodemographic characteristics and the SDOH score, with categorical variables reported as frequencies and percentages and continuous variables reported as means (SDs). Before examining associations between sociodemographic factors and the SDOH score, we first assessed the distribution of SDOH scores among respondents who completed the SDOH measures (n=149). The score ranged from 0 to 14 (15 possible values), although only 11 outcome scores were observed, with no participants reporting scores of 9, 11, 13, or 14. Table S1 in Multimedia Appendix 1 presents these observed scores and participant characteristics for each score. Given the relatively small sample size (n=149) and the number of observed outcome scores, the proportional odds assumption could be restrictive, and model estimates might be unstable because of sparse data for some scores. Guided by the score distribution shown in Table S1 in Multimedia Appendix 1, we collapsed the 11 observed outcome scores into 5 categories (0, 1, 2, 3, and 4-14) to improve model stability while preserving meaningful variability in the outcome.

The collapsed SDOH category was modeled using proportional odds logistic regression, which estimates the log-odds of being in a higher category of the ordinal outcome relative to all lower categories cumulatively. This model assumes that the association between each demographic variable and the collapsed SDOH category is constant across all outcome thresholds (the proportional odds assumption). Covariates included age, sex, years of US residence, English proficiency, education, marital status, employment status, and annual household income. Health insurance type was not included in the regression analyses because participants could select multiple categories. The model was specified as follows:


$$\log\left(\frac{\mathrm{Pr}(Y\leq k)}{\mathrm{Pr}(Y§gt;k)}\right)=\alpha_{k}+\beta^{T}X,\;k=1,\ldots ,K-1,$$

where *Y* denotes the collapsed SDOH category, and *X* denotes covariates of interest. *β* represents regression coefficients, and *α*_*k*_ represents threshold-specific intercepts. We first fitted univariable models to examine associations between each sociodemographic factor and the collapsed SDOH category. We then fitted a multivariable model including all sociodemographic factors to assess their independent associations with the collapsed SDOH category. Before conducting the analyses, the proportional odds assumption was evaluated for all ordinal regression models using the Brant test implemented in R (version 4.5.1; R Foundation for Statistical Computing), confirming that the assumption of parallel regression coefficients was reasonable for the included variables (Table S2 in Multimedia Appendix 1). The proportional odds assumption was evaluated for all ordinal regression models using the nominal effects test, and no evidence of violation was detected. Odds ratios (ORs) were obtained by exponentiating the estimated regression coefficients (exp [β]). Corresponding 95% CIs were calculated as exp(β̂±1.96×SE), and Wald tests were used to assess statistical significance. All analyses were conducted using R software with a 2-sided significance level of .05.

We also conducted univariable and multivariable regression analyses using the original SDOH score as the outcome to examine associations between sociodemographic characteristics and SDOH. Incidence rate ratios, corresponding 95% CIs, and *P* values were calculated. We conducted sensitivity analyses using a negative binomial model to evaluate the robustness of our results. The negative binomial model was used because the SDOH score is a count variable that may exhibit overdispersion, making this model appropriate for such outcomes. For univariable and multivariable analyses, we also evaluated model fit using Akaike Information Criterion (AIC) and Bayesian Information Criterion (BIC) for both the proportional odds regression model with the collapsed SDOH categories and the negative binomial regression model with the original SDOH score. Because of the small cell size for the “unreported/don’t know” household income category (10/149, 6.7%), we conducted sensitivity analyses excluding these participants and treating this response as missing rather than as a separate category. We then reran the multivariable analyses using (1) proportional odds regression with collapsed SDOH categories and (2) negative binomial regression with the original SDOH score.

## Results

### Participant Characteristics

A total of 150 participants had a mean age of 49.9 (SD 12.6) years (Table 1). Most were female (n=124, 82.7%) and born outside the United States (n=149, 99.3%), including 129 (86.0%) from mainland China and 15 (10.0%) from Hong Kong (data not shown). The mean length of US residence was 19.4 (SD 10.6) years. Overall, 132 (88.0%) participants reported speaking English less than very well, 81 (54.0%) had a high school education or less, 116 (77.3%) were married or cohabitating, 102 (68.0%) were employed, 56 (37.3%) reported an annual household income of US <$25,000, and 75 (50.0%) were insured through Medicaid.

**Table 1.** Participant characteristics of Chinese Americans at risk for diabetes (N=150).

Characteristics	Values	
Age (years), mean (SD)	49.9 (12.6)	
Female sex, n (%)	124 (82.7)	
Born outside the United States, n (%)	149 (99.3)	
Period of residence in the United States (years), mean (SD)	19.4 (10.6)	
English proficiency, n (%)		
Very well	18 (12.0)	
Well	50 (33.3)	
Not well	68 (45.3)	
Not at all	14 (9.3)	
Education, n (%)		
Less than high school	41 (27.3)	
High school graduate	40 (26.7)	
More than high school	69 (46.0)	
Marital status, n (%)		
Married or cohabitating	116 (77.3)	
Other	34 (22.7)	
Employment status, n (%)		
Employed	102 (68.0)	
Unemployed	20 (13.3)	
Retired	28 (18.7)	
Annual household income (US $), n (%)		
<25,000	56 (37.3)	
25,000-55,000	47 (31.3)	
>55,000	37 (24.7)	
Unreported/don’t know	10 (6.7)	
Insurance type^a^, n (%)		
Medicaid	75 (50.0)	
Medicare	18 (12.0)	
Private insurance	20 (13.3)	
Other insurance (public or government)	26 (17.3)	
Employee-sponsored insurance	22 (14.7)	
Uninsured	6 (4.0)	

a Participants could select multiple responses; therefore, percentages do not total 100%.

### SDOH Measures Analysis

Among the 149 respondents who completed the SDOH measures, the mean SDOH score was 2.4 (SD 2.3), and 81.9% (n=122) of participants reported at least 1 SDOH barrier (Table 2). The five most frequently reported barriers were (1) the need to improve English proficiency, reading skills, or educational attainment (n=77, 51.7%); (2) experiences of racial discrimination (n=49, 32.9%); (3) adverse housing conditions (eg, mold, pests, or structural damage; n=38, 25.5%); (4) the need for welfare support (eg, health insurance; n=26, 17.4%); and (5) concern about child behavioral problems (n=26, 17.4%). After collapsing the original SDOH scores into 5 categories, 27 (18.2%) participants had a score of 0, 39 (26.2%) had a score of 1, 23 (15.4%) had a score of 2, 27 (18.2%) had a score of 3, and 33 (22.1%) had scores of 4 to 14 (Table 3).

**Table 2.** Social determinants of health (SDOH) among respondents who completed the SDOH measures (N=149).

SDOH measures	Values	
Overall SDOH score, mean (SD)	2.4 (2.3)	
Participants reporting any SDOH-related barriers, n (%)	122 (81.9)	
Need to improve English proficiency, reading skills, or educational attainment	77 (51.7)	
Experiences of racial discrimination	49 (32.9)	
Adverse housing conditions (eg, mold, pests, or structural damage)	38 (25.5)	
Need for welfare support (eg, benefits enrollment or health insurance)	26 (17.4)	
Concern about child behavioral problems (eg, tantrums or hitting)	26 (17.4)	
Need for childcare support	23 (15.4)	
Housing insecurity or concern about eviction or utility shutoff	23 (15.4)	
Food insufficiency (food running out due to lack of money)	23 (15.4)	
Transportation barriers affecting appointments, work, or essential activities	22 (14.8)	
Worry about running out of food due to financial stress	20 (13.4)	
Need for essential child supplies (eg, diapers, car seat, crib, or stroller)	14 (9.4)	
Need for legal assistance (eg, immigration or custody)	11 (7.4)	
Need for help addressing tobacco, alcohol, or drug use	9 (6.0)	
Domestic violence–related safety concerns at home	2 (1.3)	

**Table 3.** Sociodemographic characteristics by collapsed social determinants of health (SDOH) categories among respondents who completed the SDOH measures (N=149).

Characteristics	Score 0 (n=27)	Score 1 (n=39)	Score 2 (n=23)	Score 3 (n=27)	Score 4-14 (n=33)
Age (years), mean (SD)	52.3 (11.1)	51.3 (13.3)	50.7 (11.9)	49.4 (14.3)	46.8 (12.2)
Sex, n (%)					
Female	19 (70.4)	27 (69.2)	21 (91.3)	24 (88.9)	32 (97.0)
Male	8 (29.6)	12 (30.8)	2 (8.7)	3 (11.1)	1 (3.0)
Period of US residence (years), mean (SD)	24.0 (9.2)	19.6 (10.4)	17.5 (12.1)	22.7 (11.7)	14.2 (7.5)
English proficiency, n (%)					
Very well	6 (22.2)	6 (15.4)	1 (4.3)	5 (18.5)	0 (0)
Well	11 (40.7)	12 (30.8)	6 (26.1)	9 (33.3)	12 (36.4)
Not well	8 (29.6)	16 (41.0)	13 (56.5)	12 (44.4)	18 (54.5)
Not at all	2 (7.4)	5 (12.8)	3 (13.0)	1 (3.7)	3 (9.1)
Education, n (%)					
Less than high school	4 (14.8)	12 (30.8)	11 (47.8)	5 (18.5)	9 (27.3)
High school graduate	7 (25.9)	8 (20.5)	3 (13.0)	9 (33.3)	13 (39.4)
More than high school	16 (59.3)	19 (48.7)	9 (39.1)	13 (48.1)	11 (33.3)
Marital status, n (%)					
Married or cohabitating	16 (59.3)	37 (94.9)	16 (69.6)	19 (70.4)	27 (81.8)
Other	11 (40.7)	2 (5.1)	7 (30.4)	8 (29.6)	6 (18.2)
Employment status, n (%)					
Employed	20 (74.1)	27 (69.2)	14 (60.9)	19 (70.4)	21 (63.6)
Unemployed	2 (7.4)	3 (7.7)	4 (17.4)	2 (7.4)	9 (27.3)
Retired	5 (18.5)	9 (23.1)	5 (21.7)	6 (22.2)	3 (9.1)
Annual household income (US $), n (%)					
<25,000	10 (37.0)	14 (35.9)	10 (43.5)	9 (33.3)	13 (39.4)
25,000-55,000	5 (18.5)	10 (25.6)	9 (39.1)	7 (25.9)	15 (45.5)
>55,000	11 (40.7)	10 (25.6)	4 (17.4)	8 (29.6)	4 (12.1)
Unreported/don’t know	1 (3.7)	5 (12.8)	0 (0)	3 (11.1)	1 (3.0)

### Associations Between Sociodemographic Characteristics and SDOH

In univariable regression analyses (Table 4), female sex (vs male; OR 3.77, 95% CI 1.77-8.20; *P*=.001), self-reported speaking English “not well” (vs “very well”; OR 3.35, 95% CI 1.33-8.69; *P*=.01), and being unemployed (vs employed; OR 2.62, 95% CI 1.08-6.58; *P*=.04) were associated with higher SDOH score categories (indicating more barriers), while longer residence in the United States (OR 0.96, 95% CI 0.94-0.99; *P*=.007) was associated with lower SDOH score categories. In multivariable analyses, female sex (OR 3.83, 95% CI 1.65-9.16; *P*=.002) remained independently associated with higher SDOH score categories.

**Table 4.** Associations between sociodemographic characteristics and collapsed social determinants of health categories.

Characteristics	Univariable analysis			Multivariable analysis	
	OR^a^ (95% CI)		*P* value	OR (95% CI)	*P* value
Age (years)	0.98 (0.96-1.01)		.08	0.97 (0.94-1.01)	.08
Female (reference: male)	3.77^b^ (1.77-8.20)		.001	3.83^b^ (1.65-9.16)	.002
Period of residence in the United States (years)	0.96^b^ (0.94-0.99)		.007	0.97 (0.94-1.01)	.14
English proficiency (reference: very well)					
Well	2.39 (0.91-6.41)		.08	1.68 (0.56-5.17)	.36
Not well	3.35^c^ (1.33-8.69)		.01	2.38 (0.62-9.26)	.21
Not at all	2.13 (0.63-7.39)		.23	2.69 (0.45-16.14)	.28
Education (reference: less than high school)					
High school graduate	1.38 (0.63-2.99)		.41	1.50 (0.63-3.59)	.36
More than high school	0.68 (0.34-1.30)		.25	1.07 (0.41-2.82)	.90
Marital status: other (reference: married or cohabitating)	0.80 (0.39-1.60)		.52	0.76 (0.35-1.64)	.49
Employment status (reference: employed)					
Unemployed	2.62^c^ (1.08-6.58)		.04	2.27 (0.89-6.07)	.09
Retired	0.82 (0.40-1.68)		.58	1.60 (0.60-4.29)	.34
Annual household income (US $; reference: <25,000)					
25,000-55,000	1.57 (0.78-3.16)		.21	1.27 (0.53-3.09)	.59
>55,000	0.55 (0.26-1.16)		.12	0.78 (0.25-2.41)	.67
Unreported/don’t know	0.77 (0.24-2.45)		.66	0.91 (0.22-3.74)	.90

a OR: odds ratio.

b *P*<.01.

c *P*<.05.

### Sensitivity Analyses

Using the original SDOH score as the outcome, Table S3 in Multimedia Appendix 1 shows that the associations between demographic variables and SDOH were generally consistent in direction and magnitude with those observed in the primary analyses. In the multivariable negative binomial model, female participants had significantly higher SDOH scores than male participants (incidence rate ratio 2.33, 95% CI 1.46-3.70; *P*<.001).

Table S4 in Multimedia Appendix 1 presents the AIC and BIC values for the proportional odds logistic regression models with collapsed SDOH categories and the negative binomial regression models with the original SDOH score. Across both univariable and multivariable analyses, the proportional odds models with collapsed SDOH categories generally showed lower AIC and BIC values than the negative binomial models, suggesting better model fit. For example, in the multivariable analysis, the AIC and BIC for the proportional odds model were 475.80 and 529.87, respectively, compared with 595.58 and 643.65 for the negative binomial model. These findings suggest that the proportional odds models provided a more parsimonious fit to the data while adequately capturing variation in SDOH outcomes.

Table S5 in Multimedia Appendix 1 shows that, when the “unreported/don’t know” household income group (10/149, 6.7%) was excluded from the multivariable analyses, the findings remained similar to those from the primary analyses. Specifically, the direction, magnitude, and statistical interpretation of the associations between sociodemographic factors and both the collapsed SDOH categories and the original SDOH score were largely unchanged. These results further support the robustness of the study findings.

## Discussion

### Principal Findings

This study examined SDOH in a NYC community-based sample of Chinese Americans at risk for diabetes. It is among the first studies to assess SDOH in this understudied population in diabetes research [27]. The “model minority” stereotype often portrays Chinese Americans as uniformly advantaged, self-sufficient, and living under relatively favorable socioeconomic and health circumstances [28]. Our findings challenge this narrative by revealing significant socioeconomic disadvantage and multiple SDOH barriers in this sample, underscoring that Chinese Americans at risk for diabetes are not a uniformly advantaged group.

Most participants (122/149, 81.9%) reported at least 1 SDOH barrier. This finding is consistent with that of a prior study of safety-net patients with uncontrolled diabetes, in which 92% of participants reported at least 1 SDOH-related barrier [29]. The most commonly reported barriers included needs related to improving English proficiency, reading skills, or educational attainment (77/149, 51.7%), experiences of racial discrimination (49/149, 32.9%), adverse housing conditions (38/149, 25.5%), the need for welfare support (26/149, 17.4%), and concerns about child behavioral problems (26/149, 17.4%). More than one-fifth of participants (33/149, 22.1%) reported 4 or more SDOH barriers. These findings highlight the multiple social and structural challenges faced by this group and suggest that diabetes prevention programs may benefit from routine SDOH screening combined with structured resource navigation and referral pathways, such as access to health-related social services. These results further challenge prevailing “model minority” narratives and underscore the heterogeneity of needs within this population [21,30]. Without data of this type, the challenges faced by many Chinese Americans may remain obscured, potentially contributing to the underallocation of resources and an increased risk of progression from prediabetes to diabetes [31].

We also found that female sex was associated with more SDOH barriers. One possible explanation is that women may experience greater barriers because of caregiving responsibilities and related constraints on employment [32] and access to other resources. However, the male reference group in this study was small (n=26), which may have reduced the precision of comparisons by sex. Accordingly, the mechanisms underlying the higher SDOH scores observed among female participants should be interpreted cautiously and warrant further investigation in larger samples. Therefore, diabetes prevention programs serving Chinese Americans may benefit from targeted support for subgroups facing higher risk, such as women.

The associations of English proficiency, length of US residence, and employment status with SDOH barriers observed in univariable analyses were attenuated in the multivariable model. This may reflect confounding and shared variance with other sociodemographic variables. Further research in larger samples is warranted to examine their independent associations with SDOH among Chinese Americans at risk for diabetes.

### Limitations

This study has several limitations. First, as an exploratory analysis of data from a single clinical trial, participants were recruited in NYC and were predominantly born outside the United States, which may limit generalizability to US-born Chinese Americans and to individuals living in other geographic regions. However, this concern is partly mitigated by the relatively long mean duration of US residence (19.4, SD 10.6 years) among participants born outside the United States. Second, the cross-sectional nature of the baseline data precludes causal inference, and self-reported measures are subject to recall and reporting bias. Finally, the modest sample size precluded domain-specific analyses of SDOH. Despite these limitations, this study provides a comprehensive assessment of SDOH in an understudied population at elevated risk for diabetes. Additionally, community-based recruitment complements health care–based samples and provides community-grounded insights to inform tailored strategies for diabetes prevention and care for Chinese Americans.

### Conclusions

SDOH-related barriers are prevalent among Chinese Americans at risk for diabetes. Findings from this NYC community–based sample have important implications for diabetes prevention and care. Programs serving this population should not rely on “model minority” assumptions and may benefit from integrating routine SDOH screening with structured resource navigation and referral pathways to address identified needs. Targeted supports may be particularly important for subgroups facing greater risk, including female individuals. Future studies in larger Chinese American populations are needed to confirm these findings.

## Supplementary material

10.2196/95295 Multimedia Appendix 1 Supplementary tables.

## Abbreviations

AIC: Akaike Information Criterion
BIC: Bayesian Information Criterion
INCLUDE: Integrating Cultural Aspects into Diabetes Education
NYC: New York City
OR: odds ratio
RA: research assistant
SDOH: social determinants of health

## References

[1] Cefalu WT, Petersen MP, Ratner RE (2014). The alarming and rising costs of diabetes and prediabetes: a call for action!. Diabetes Care.

[2] Dall TM, Zhang Y, Chen YJ, Quick WW, Yang WG, Fogli J (2010). The economic burden of diabetes. Health Aff (Millwood).

[3] Dall TM, Yang W, Halder P (2014). The economic burden of elevated blood glucose levels in 2012: diagnosed and undiagnosed diabetes, gestational diabetes mellitus, and prediabetes. Diabetes Care.

[4] Tabák AG, Herder C, Rathmann W, Brunner EJ, Kivimäki M (2012). Prediabetes: a high-risk state for diabetes development. Lancet.

[5] National diabetes statistics report. https://www.cdc.gov/diabetes/php/data-research/index.html.

[6] Nathan DM, Davidson MB, DeFronzo RA (2007). Impaired fasting glucose and impaired glucose tolerance: implications for care. Diabetes Care.

[7] Social determinants of health. World Health Organization.

[8] Social determinants of health. U.S. Department of Health and Human Services.

[9] Frier A, Devine S, Barnett F, McBain-Rigg K, Dunning T (2022). Improving type 2 diabetes care and self-management at the individual level by incorporating social determinants of health. Aust N Z J Public Health.

[10] Woodward A, Walters K, Davies N (2024). Barriers and facilitators of self-management of diabetes amongst people experiencing socioeconomic deprivation: a systematic review and qualitative synthesis. Health Expect.

[11] Hu J, Amirehsani K, Wallace DC, Letvak S (2013). Perceptions of barriers in managing diabetes: perspectives of Hispanic immigrant patients and family members. Diabetes Educ.

[12] Martinez-Cardoso A, Jang W, Baig AA (2020). Moving diabetes upstream: the social determinants of diabetes management and control among immigrants in the US. Curr Diab Rep.

[13] Hacker K, Thomas CW, Zhao G, Claxton JS, Eke P, Town M (2024). Social determinants of health and health-related social needs among adults with chronic diseases in the United States, Behavioral Risk Factor Surveillance System, 2022. Prev Chronic Dis.

[14] McClintock HF, Edmonds SE, Wang E (2026). Racial/ethnic disparities in access to transportation among persons with type 2 diabetes mellitus. J Racial Ethn Health Disparities.

[15] Hill-Briggs F, Fitzpatrick SL (2023). Overview of social determinants of health in the development of diabetes. Diabetes Care.

[16] (2023). 2023 ACS 1-year estimates. United States Census Bureau.

[17] (2025). Facts about Chinese in the U.S. Pew Research Center.

[18] Vicks WS, Lo JC, Guo L (2022). Prevalence of prediabetes and diabetes vary by ethnicity among U.S. Asian adults at healthy weight, overweight, and obesity ranges: an electronic health record study. BMC Public Health.

[19] Greene M, Batalova J (2025). Chinese immigrants in the United States. Migration Policy Institute.

[20] Wang A (2022). Chinese in NYC: a profile. Asian American Federation.

[21] Tan C, Wyatt LC, Kranick JA, Kwon SC, Oyebode O (2018). Factors associated with health insurance status in an Asian American population in New York City: analysis of a community-based survey. J Racial Ethn Health Disparities.

[22] Hu L, Lin NF, Shi Y (2024). The Integrating Cultural Aspects Into Diabetes Education (INCLUDE) study to prevent diabetes in Chinese immigrants: protocol for a randomized controlled trial. JMIR Res Protoc.

[23] Bang H, Edwards AM, Bomback AS (2009). Development and validation of a patient self-assessment score for diabetes risk. Ann Intern Med.

[24] WHO Expert Consultation (2004). Appropriate body-mass index for Asian populations and its implications for policy and intervention strategies. Lancet.

[25] Hager ER, Quigg AM, Black MM (2010). Development and validity of a 2-item screen to identify families at risk for food insecurity. Pediatrics.

[26] The Accountable Health Communities Health-Related Social Needs Screening Tool. Centers for Medicare & Medicaid Services.

[27] Rajpathak SN, Wylie-Rosett J (2011). High prevalence of diabetes and impaired fasting glucose among Chinese immigrants in New York City. J Immigr Minor Health.

[28] Yi SS, Kwon SC, Sacks R, Trinh-Shevrin C (2016). Commentary: persistence and health-related consequences of the model minority stereotype for Asian Americans. Ethn Dis.

[29] Levy NK, Park A, Solis D (2022). Social determinants of health and diabetes-related distress in patients with insulin-dependent type 2 diabetes: cross-sectional, mixed methods approach. JMIR Form Res.

[30] Chesla CA, Chun KM, Kwan CM (2013). Testing the efficacy of culturally adapted coping skills training for Chinese American immigrants with type 2 diabetes using community-based participatory research. Res Nurs Health.

[31] Min LY, Islam RB, Gandrakota N, Shah MK (2022). The social determinants of health associated with cardiometabolic diseases among Asian American subgroups: a systematic review. BMC Health Serv Res.

[32] Pendergrast C (2021). Women report worse employment impacts from family caregiving. Maxwell School of Citizenship and Public Affairs, Syracuse University.

