# Supplementary material for: Social Determinants of Health Among Chinese Americans at Risk for Diabetes in a Mobile Diabetes Prevention Trial: Cross-Sectional Baseline Analysis
Source: JMIR Diabetes. 2026 Jun 3;11:e95295. doi: 10.2196/95295 (PMC13232781; doi:10.2196/95295)
Supplement: Multimedia Appendix 1 [file diabetes-v11-e95295-s001.docx]

Table S1: Observed social determinants of health categories among respondents to the measures (N = 149)

|  | Score 0  (n=27; 18.2%) | Score 1  (n=39; 26.2%) | Score 2  (n=23; 15.4%) | Score 3  (n=27; 18.2%) | Score 4  (n=8; 5.4%) | Score 5  (n=8; 5.4%) | Score 6  (n=7; 4.7%) | Score 7  (n=4; 2.7%) | Score 8  (n=3; 2.0%) | Score 10  (n=2; 1.3%) | Score 12  (n=1; 0.7%) |
| --- | --- | --- | --- | --- | --- | --- | --- | --- | --- | --- | --- |
|  | n (%) | n (%) | n (%) | n (%) | n (%) | n (%) | n (%) | n (%) | n (%) | n (%) | n (%) |
| Age, mean (SD) | 52.3 (11.1) | 51.3 (13.3) | 50.7 (11.9) | 49.4 (14.3) | 45.5 (11.4) | 42.6 (12.3) | 55.6 (14.9) | 38.0 (1.2) | 53.3 (7.4) | 49.5 (13.4) | 41.0 |
| Sex |  |  |  |  |  |  |  |  |  |  |  |
| Female | 19 (70.4) | 27 (69.2) | 21 (91.3) | 24 (88.9) | 7 (87.5) | 8 (100) | 7 (100) | 4 (100) | 3 (100) | 2 (100) | 1 (100) |
| Male | 8 (29.6) | 12 (30.8) | 2 (8.7) | 3 (11.1%) | 1 (12.5) | 0 | 0 | 0 | 0 | 0 | 0 |
| Years of U.S. residence, mean (SD) | 24.0 (9.2) | 19.6 (10.4) | 17.5 (12.1) | 22.7 (11.7) | 13.4 (8.6) | 12.0 (3.3) | 18.1 (7.7) | 17.3 (8.9) | 16.7 (9.1) | 6.00 (5.7) | 9.0 |
| English proficiency |  |  |  |  |  |  |  |  |  |  |  |
| Very well | 6 (22.2) | 6 (15.4) | 1 (4.3) | 5 (18.5) | 0 | 0 | 0 | 0 | 0 | 0 | 0 |
| Well | 11 (40.7) | 12 (30.8) | 6 (26.1) | 9 (33.3) | 5 (62.5) | 2 (25.0) | 3 (42.9) | 2 (50.0) | 0 | 0 | 0 |
| Not well | 8 (29.6) | 16 (41.0) | 13 (56.5) | 12 (44.4) | 3 (37.5) | 5 (62.5) | 3 (42.9) | 2 (50.0) | 3 (100) | 1 (50.0) | 1 (100) |
| Not at all | 2 (7.4) | 5 (12.8) | 3 (13.0) | 1 (3.7) | 0 | 1 (12.5) | 1(14.3) | 0 | 0 | 1 (50.0) | 0 |
| Education |  |  |  |  |  |  |  |  |  |  |  |
| Less than high school | 4 (14.8) | 12 (30.8) | 11 (47.8) | 5 (18.5) | 3 (37.5) | 2 (25.0) | 2 (28.6) | 0 | 1 (33.3) | 1 (50.0) | 0 |
| High school graduate | 7 (25.9) | 8 (20.5) | 3 (13.0) | 9 (33.3) | 2 (25.0) | 4 (50.0) | 3 (42.9) | 2 (50.0) | 1 (33.3) | 1 (50.0) | 0 |
| More than high school | 16 (59.3) | 19 (48.7) | 9 (39.1) | 13 (48.1) | 3 (37.5) | 2 (25.0) | 2 (28.6) | 2 (50.0) | 1 (33.3) | 0 | 1 (100) |
| Marital status |  |  |  |  |  |  |  |  |  |  |  |
| Married or cohabitating | 16 (59.3) | 37 (94.9) | 16 (69.6) | 19 (70.4) | 7 (87.5) | 6 (75.0) | 5 (71.4) | 3 (75.0%) | 3 (100) | 2 (100) | 1 (100) |
| Other | 11 (40.7) | 2 (5.1) | 7 (30.4) | 8 (29.6) | 1 (12.5) | 2 (25.0) | 2 (28.6) | 1 (25.0%) | 0 | 0 | 0 |
| Employment status |  |  |  |  |  |  |  |  |  |  |  |
| Employed | 20 (74.1) | 27 (69.2) | 14 (60.9) | 19 (70.4) | 8 (100) | 3 (37.5) | 4 (57.1) | 2 (50.0) | 2 (66.7) | 1 (50.0) | 1 (100) |
| Unemployed | 2 (7.4) | 3 (7.7) | 4 (17.4) | 2 (7.4) | 0 | 4 (50.0) | 1 (14.3) | 2 (50.0) | 1 (33.3) | 1 (50.0) | 0 |
| Retired | 5 (18.5) | 9 (23.1) | 5 (21.7) | 6 (22.2) | 0 | 1 (12.5) | 2 (28.6) | 0 | 0 | 0 | 0 |
| Annual household income |  |  |  |  |  |  |  |  |  |  |  |
| < $25,000 | 10 (37.0) | 14 (35.9) | 10 (43.5) | 9 (33.3) | 2 (25.0) | 4 (50.0) | 3 (42.9) | 1 (25.0) | 2 (66.7) | 1 (50.0) | 0 |
| $25,000 - $55,000 | 5 (18.5) | 10 (25.6) | 9 (39.1) | 7 (25.9) | 5 (62.5) | 4 (50.0) | 1 (14.3) | 2 (50.0) | 1 (33.3) | 1 (50.0) | 1 (100) |
| > $55,000 | 11 (40.7) | 10 (25.6) | 4 (17.4) | 8 (29.6) | 1 (12.5) | 0 | 2 (28.6) | 1 (25.0) | 0 | 0 | 0 |
| Unreported/Don’t know | 1 (3.7) | 5 (12.8) | 0 | 3 (11.1) | 0 | 0 | 1 (14.3) | 0 | 0 | 0 | 0 |

Table S2: Brant test p-values for the proportional odds assumption in univariable and multivariable proportional logistical regression models using the collapsed SDOH category as the outcome

|  | *P* values in univariable regression models | *P* values in multivariable regression models |
| --- | --- | --- |
| Age | 0.96 | 0.89 |
| Male (vs. Female) | 0.39 | 0.6 |
| Years of U.S. residence | 0.04 | 0.07 |
| English proficiency (ref: very well) |  |  |
| Well | 0.88 | 0.80 |
| Not Well | 0.44 | 0.78 |
| Not at all | 0.63 | 0.93 |
| Education (ref: Less than high school) |  |  |
| High school graduate | 0.05 | 0.08 |
| More than high school | 0.19 | 0.64 |
| Marital status (ref: married/cohabitating) |  |  |
| Other | < 0.01 | < 0.01 |
| Employment status (ref: employed) |  |  |
| Unemployed | 0.42 | 0.82 |
| Retired | 0.70 | 0.79 |
| Annual household income (ref: < $25,000) |  |  |
| $25,000 - $55,000 | 0.96 | 0.68 |
| > $55,000 | 0.56 | 0.87 |
| Unreported/Don’t know | < 0.01 | 0.17 |

Table S3. Associations between sociodemographic characteristics and the original SDOH score using negative binomial

|  | Univariable Analysis | | |  | Multivariable Analysis | | |
| --- | --- | --- | --- | --- | --- | --- | --- |
|  | IRR | [95% CI] | *P* |  | IRR | [95% CI] | *P* |
| Age | 0.99 | [0.98, 1.00] | 0.104 |  | 0.99 | [0.97, 1.01] | 0.196 |
| Female (vs. male) | 2.44^***^ | [1.54, 3.87] | < 0.001 |  | 2.33^***^ | [1.46, 3.70] | < 0.001 |
| Years of residence in the U.S. | 0.98 | [0.96, 0.99] | 0.002 |  | 0.99 | [0.97, 1.00] | 0.112 |
| English proficiency (ref: very well) |  |  |  |  |  |  |  |
| Well | 1.77^*^ | [1.00, 3.12] | 0.049 |  | 1.40 | [0.79, 2.49] | 0.252 |
| Not well | 2.25^**^ | [1.30, 3.90] | 0.004 |  | 1.90 | [0.98, 3.67] | 0.056 |
| Not at all | 1.96 | [0.98, 3.91] | 0.057 |  | 2.11 | [0.89, 5.02] | 0.092 |
| Education (ref: less than high school) | |  |  |  |  |  |  |
| High school graduate | 1.21 | [0.82, 1.79] | 0.348 |  | 1.31 | [0.90, 1.90] | 0.154 |
| More than high school | 0.86 | [0.60, 1.24] | 0.414 |  | 1.19 | [0.78, 1.82] | 0.417 |
| Marital status (ref: married/cohabitating) | |  |  |  |  |  |  |
| Other | 0.85 | [0.59, 1.22] | 0.379 |  | 0.80 | [0.56, 1.14] | 0.223 |
| Employment status (ref: employed) |  |  |  |  |  |  |  |
| Unemployed | 1.61^*^ | [1.07, 2.42] | 0.021 |  | 1.44 | [0.99, 2.09] | 0.059 |
| Retired | 0.83 | [0.55, 1.24] | 0.359 |  | 1.06 | [0.67, 1.68] | 0.812 |
| Annual household income (ref: < $25,000) | | |  |  |  |  |  |
| $25,000 - $55,000 | 1.21 | [0.86, 1.71] | 0.283 |  | 1.01 | [0.69, 1.46] | 0.969 |
| > $55,000 | 0.70 | [0.47, 1.05] | 0.083 |  | 0.87 | [0.52, 1.45] | 0.597 |
| Unreported/Don’t know | 0.80 | [0.42, 1.52] | 0.495 |  | 0.93 | [0.49, 1.78] | 0.835 |
| *Notes.* IRR: Incidence rate ratio; CI: Confidence interval.  ^*^ *P* < .05; ^**^ *P* < .01; ^***^ *P* < .001. | | | | | | | |

Table S4: Model fit statistics (AIC and BIC) for proportional odds model with the collapsed SDOH category and negative model with original count-based SDOH scores

|  |  | Proportional regression model  with collapsed SDOH category | | Negative binomial model  with original SDOH score | |
| --- | --- | --- | --- | --- | --- |
|  |  | AIC | BIC | AIC | BIC |
| Bivariable | Age | 481.32 | 496.34 | 608.78 | 617.80 |
|  | Gender | 472.46 | 487.48 | 596.61 | 605.62 |
|  | Years of U.S. residence | 477.00 | 492.02 | 601.61 | 610.62 |
|  | Education | 482.3 | 500.33 | 609.93 | 621.95 |
|  | Annual household income | 481.38 | 502.4 | 608.25 | 623.27 |
|  | Marital status | 484.06 | 499.08 | 610.63 | 619.64 |
|  | Employment status | 480.98 | 499.00 | 606.18 | 618.20 |
|  | English proficiency | 481.66 | 502.69 | 606.28 | 621.30 |
| Multivariable | **-** | 475.8 | 529.87 | 595.58 | 643.65 |

Table S5: Sensitivity analysis: logistic regression of the association between sociodemographic factors and the collapsed SDOH categories and original SDOH scores without the “Unreported/Don’t know” household income group

|  | Proportional odds model with  collapsed SDOH category | | |  | Negative binomial model with  original count-based SDOH score | | |
| --- | --- | --- | --- | --- | --- | --- | --- |
|  | OR | *P* | 95% CI |  | IRR | *P* | 95% CI |
| Age | 0.97 | 0.86 | [0.93, 1.00] |  | 0.99 | 0.226 | [0.97, 1.01] |
| Female (vs. Male) | 4.08^**^ | 0.002 | [1.68, 10.15] |  | 2.50^***^ | < 0.001 | [1.52, 4.09] |
| Years of U.S. residence | 0.97 | 0.119 | [0.94, 1.01] |  | 0.99 | 0.094 | [0.97, 1.00] |
| English proficiency (ref: very well) |  |  |  |  |  |  |  |
| Well | 1.65 | 0.376 | [0.54, 5.11] |  | 1.37 | 0.281 | [0.64, 1.64] |
| Not well | 2. 52 | 0.202 | [0.61, 10.54] |  | 1.92 | 0.059 | [0.77, 2.44] |
| Not at all | 2.83 | 0.268 | [0.45, 10.01] |  | 2.17 | 0.086 | [0.90, 5.25] |
| Education (ref: Less than high school) |  |  |  |  |  |  |  |
| High school graduate | 1.44 | 0.422 | [0.59, 3.56] |  | 1.30 | 0.175 | [0.89, 1.90] |
| More than high school | 1.27 | 0.658 | [0.44, 3.66] |  | 1.29 | 0.261 | [0.83, 2.02] |
| Marital status (ref: married/cohabitating) |  |  |  |  |  |  |  |
| Other | 0.60 | 0.213 | [0.26, 1.34] |  | 0.71 | 0.076 | [0.49, 1.04] |
| Employment status (ref: employed) |  |  |  |  |  |  |  |
| Unemployed | 2.19 | 1.09 | [0.85, 5.89] |  | 1.43 | 0.061 | [0.98, 2.08] |
| Retired | 1.45 | 0.470 | [0.53, 3.97] |  | 1.02 | 0.934 | [0.64, 1.64] |
| Annual household income (ref: < $25,000) |  |  |  |  |  |  |  |
| $25,000 - $55,000 | 1.10 | 0.836 | [0.45, 2.68] |  | 0.95 | 0.781 | [0.65, 1.38] |
| > $55,000 | 0.66 | 0.479 | [0.21, 2.07] |  | 0.82 | 0.439 | [0.49, 1.37] |
| *Notes.* OR: Odds ratio; IRR: Incidence rate ratio; CI: Confidence interval.  ^*^ *P* < .05; ^**^ *P* < .01; ^***^ *P* < .001 | | | | | | | |
